# Understanding Odor Information Segregation in the Olfactory Bulb by Means of Mitral and Tufted Cells

**DOI:** 10.1371/journal.pone.0109716

**Published:** 2014-10-30

**Authors:** Davide Polese, Eugenio Martinelli, Santiago Marco, Corrado Di Natale, Agustin Gutierrez-Galvez

**Affiliations:** 1 Istituto per la Microelettronica e Microsistemi, Consiglio Nazionale delle Ricerche, Roma, Italy; 2 University of Rome Tor Vergata, Electronic Engineering Department, Roma, Italy; 3 Institute for Bioengineering of Catalonia, Barcelona, Spain; 4 Universitat de Barcelona, Electronics Department, Barcelona, Spain; Université Lyon, France

## Abstract

Odor identification is one of the main tasks of the olfactory system. It is performed almost independently from the concentration of the odor providing a robust recognition. This capacity to ignore concentration information does not preclude the olfactory system from estimating concentration itself. Significant experimental evidence has indicated that the olfactory system is able to infer simultaneously odor identity and intensity. However, it is still unclear at what level or levels of the olfactory pathway this segregation of information occurs. In this work, we study whether this odor information segregation is performed at the input stage of the olfactory bulb: the glomerular layer. To this end, we built a detailed neural model of the glomerular layer based on its known anatomical connections and conducted two simulated odor experiments. In the first experiment, the model was exposed to an odor stimulus dataset composed of six different odorants, each one dosed at six different concentrations. In the second experiment, we conducted an odor morphing experiment where a sequence of binary mixtures going from one odor to another through intermediate mixtures was presented to the model. The results of the experiments were visualized using principal components analysis and analyzed with hierarchical clustering to unveil the structure of the high-dimensional output space. Additionally, Fisher's discriminant ratio and Pearson's correlation coefficient were used to quantify odor identity and odor concentration information respectively. Our results showed that the architecture of the glomerular layer was able to mediate the segregation of odor information obtaining output spiking sequences of the principal neurons, namely the mitral and external tufted cells, strongly correlated with odor identity and concentration, respectively. An important conclusion is also that the morphological difference between the principal neurons is not key to achieve odor information segregation.

## Introduction

The olfactory system of animals is continuously exposed to a large variety of odor stimuli at changing concentrations. In spite of this variability of conditions, it identifies odorants with great robustness. This is due to its capability to transform these environmental stimuli into odor representations of spatiotemporal neural activity that are invariant to odor concentration [1], [2]. This perceptual ability, however, does not preclude the olfactory system to recognize the concentration at which odors are presented. It is apparent that the olfactory system is able to develop along with a concentration-invariant neural representation another, yet unveiled, neural representation that encodes for odor concentration [3], [4], [5], [6]. To date, the coding mechanism and the anatomical location that mediates this segregation of information are still unknown.

Olfactory coding starts at the olfactory epithelium when airborne odor molecules enter into the nasal cavity and interact with the olfactory receptors (OR). OR are located on the cilia of the olfactory receptor neurons (ORNs) that extent on the surface of the olfactory epithelium. Each ORN expresses a single OR type that is able to interact with many different odorant molecules, at the same time, each odorant can bind to several OR types. This give rise to a combinatorial code that captures odor identity information as patterns of activation across ORNs [7], [8]. Beyond the olfactory epithelium, the ORNs project their axons into spherical areas of the olfactory bulb (OB) called glomeruli, where each glomerulus selectively receives axons from ORNs expressing the same OR [9], [10]. In the OB, odor stimulation evokes odor-specific temporal patterns of activity at the levels of both, the olfactory nerve inputs (activation of glomeruli) and outputs (projection neurons) [11]. These complex spiking patterns are then sent to the olfactory (piriform) cortex where odor information is thought to be decoded [11], [12], [13].

The internal synaptic arrangement found among glomeruli in the OB has been deeply investigated over the past years [11], [14], [15]. Although the functional relevance of this synaptic arrangement remains to be established, some neuronal microcircuits of the glomerulus and/or OB have been considered to play a critical role in the olfactory bulb's information processing [11]. Specifically, the ORN → external tufted (ET) → short axon (SA) interconnection arrangement is thought to be responsible for pattern normalization and a first level of contrast enhancement [16], [17]. Whereas the Mitral (MC) → Granular (GC) → MC arrangement is thought to mediate a second level of contrast enhancement [18], [19]. Additionally, the presence of two different projection neurons as output of the OB, and the different behavior between MCs and ETs have led to the idea that these neurons play also different roles in the processing of olfactory information [13], [20], [21] that may be related to the coding of odor intensity and identity. However, whether and how the concentration information processing occurs at these stages and, in particular, the exact role of the MCs and ET cells in this respect remains unknown.

**Figure 1:**
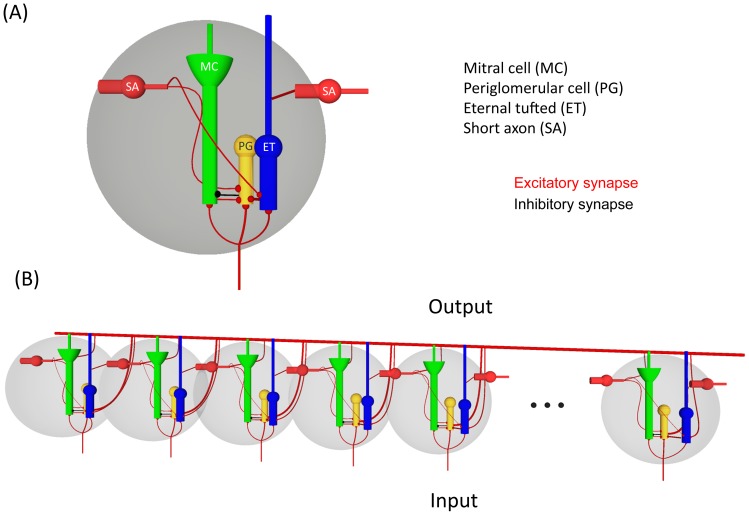
Glomerular layer model. (A) 16 glomerulus connected through SAs. The thick red line represents full connectivity between glomerulus. b) Architecture of the glomerular unit. The cells depicted are: mitral cell (MC), external tufted cell (ET), periglomerular cell (PG), and short axon (SA). Black balls represent inhibitory synapses and red balls indicate excitatory synapses. The ORN input synapses into MC, ET and PG cells. MC and PG cells form a negative feedback loop, where PG cells inhibit MC cells and in turn MC excite PG cells. ET cells contribute to the inhibition of MC through an excitatory connection to PG cells. Finally, connections between glomerulus are achieved via SA cells, which receive excitatory inputs from ET cells and transmit its outputs to PG and ET in an excitatory fashion.

In this work, we tested the hypothesis that MCs and ETs are responsible for coding odor identity information and odor concentration information respectively. To this end, we developed a computational model of the glomerular layer based on their known anatomical and morphological characteristics (Figure 1). To test our hypothesis, the computational model was exposed to two odor experiments including pure odors dosed at different concentrations and binary odor mixtures. The output signals of the model were analyzed using statistical methods to determine the structure of the high-dimensional output space and to quantify the odor identity and odor concentration information.

## Materials and Methods

### The Odor stimuli

The objective of this work is to analyze the glomerular layer ability to enhance the classification of distinct odorants (i.e., odor class identification) and the estimation of odor concentrations (i.e., odor quantification). We consider that the role of ORNs to develop these tasks is not critical for the glomerular mechanisms and is outside the scope of this study. For these reasons, there is no explicit ORN model used to provide the glomerular input. Instead, to generate this input, we have considered the combinatorial code strategy [7] along with the fact that each glomerulus receives axons from ORNs expressing the same receptor protein. Bearing this in mind, we characterize an odorant as a pattern of activation across glomeruli. Where this pattern reflects the affinity to this odorant of the receptors corresponding to each glomerulus. In our model, the input to the glomeruli is introduced as a constant current injected to the different neurons. Thus, the activation pattern representing each odorant is a current vector. The model was formed by 16 glomeruli each representing a different kind of ORN.

**Figure 2:**
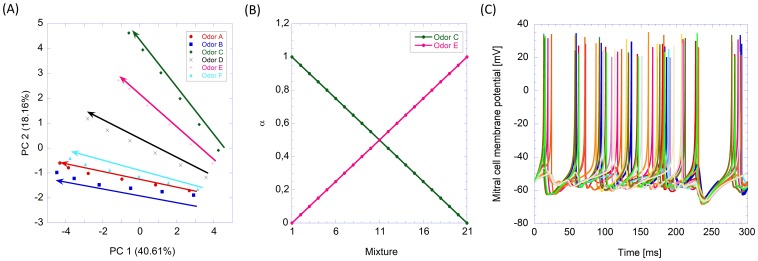
Input odor patters and MC output activity. (A) Scores plot of the first two principal components of the input odors used to analyze the glomerular layer model. The directions of the arrows indicate increasing concentration. (B) Relative composition of odor C and E on the series of binary mixtures that simulate the morphing between the two odors. The y-axis shows the relative composition of odor C and odor E in each one of the 21 mixtures. (C) Example of mitral cell responses of a 16-glomeruli model. Different colors identify different mitral cells.

In the first experiment, we exposed the glomerular model to 6 odorants at 6 different concentrations each. We assumed that each of the 16 classes of ORNs is sensitive to all the odorants but with different magnitudes, furthermore a linear relationship between concentration and response exists. Then the response of each class of ORN, input to its relevant glomerulus, is R_ORN_^odor^  =  S_ORN_C^odor^. The quantities S_ORN_ were randomly generated from a uniform distribution ranging from 0 pA to 40 pA. Concentrations (C^odor^) are dimensionless quantities in the range {0.4, 0.6, 0.8, 1, 1.2, 1.4}. Eventually, 36 vectors of responses were generated. The magnitude of the response pattern is limited to 40 pA, taking into account the saturation effect of the ORN response. The 36 vectors are shown in figure 2a.

In the second experiment, we exposed a 16 glomerular model to a series of 21 binary mixtures. This series mimics the slow evolution of the odor pattern from odor C to odor E (first experiment) both with a concentration factor of 1. The odor patterns of the binary mixtures were obtained as a convex combination of the constituent odorants.
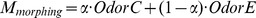
(1)where α takes 21 values distributed uniformly from the range [0, 1] with a step of 0.05 (figure 2b).

### Neuron Model

The glomerular layer is a complex system that is made up of a large number of neurons and interconnections, including MC, ET, periglomerular (PG), and short axon cells, which are morphologically different. The neurons of the glomerular layer were modeled by means of the Izhikevich's model [22], a spiking neuron model capable of reproducing the spiking and bursting behavior of different types of neurons. It is able to reduce the complexity of Hodgking-Huxley neural models maintaining its biological plausibility. Its behavior obeys the following two-dimensional system of ordinary differential equations:
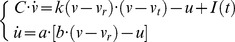
(2)
*s.t.* the following after-spike reset condition:
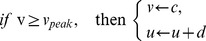
(3)where the variables *v* and *u* are the neuron membrane potential and the membrane recovery variable, respectively. The parameters in equations 2 and 3 can be interpreted as follows: *C* is the membrane capacitance, *v_r_* is the resting membrane potential (i.e. the membrane voltage at which the network membrane current is equal to zero), *v_t_* is the instantaneous threshold potential, *v_peak_* is the spike peak voltage, *a* is the recovery time constant, *b* is the recovery variable depending on the sub-threshold fluctuations of the membrane potential, *c* is the after-spike reset value of the membrane potential, *d* describes the after-spike reset of the recovery variable, and *I(t)* represents the total input current on the membrane neuron. For further details about this model the reader is referred to [22].

Table 1 illustrates the parameters used to implement the different neurons utilized in our model, which were chosen based on previous experimental evidence to match the experimental frequency-current responses. Specifically, the parameters of the MCs and ET cells were selected by following data reported in [22]. The parameters of PG cells were obtained from [23], [24], whereas the SA cell parameters were taken from [25]. Figure 2c shows the time evolution of the membrane potential of the MC obtained with the Izhikevich's model and the parameters of Table 1 of a 16-glomeruli model.

**Table 1:** Model parameters of the olfactory bulb neurons.

*Neuron\Parameter*	*C[pF]*	*k[GΩ^−1^mV^−1^]*	*v_r_[mV]*	v_t_[mV]	*a [ms^−1^]*	*b[GΩ^−1^]*	*v_peak_[mV]*	c[mV]	*d[mV]*
*Mitral cell*	*40*	*1*	*−55*	−50	*0.4*	*2.6*	*35*	−50	*200*
*Periglomerular*	*5.9*	*0.049*	*−53.1*	−20	*0.0167*	*−0.94*	*35*	−20	*50*
*Tufted cell*	*40*	*1*	*−55*	−50	*0.4*	*2.6*	*35*	−50	*200*
*Short axon cell*	*58*	*0.061*	*−67*	−30	*0.049*	*−0.68*	*35*	−30	*150*

### Network Model

The interconnections between neurons in our model follow those found in the glomerular layer as described in [16], [17] and [26]. The glomerular model is built as an ensemble of interconnected glomerulus, which are composed of four different types of neurons, including two principal neurons called MCs and ET cells, and two interneurons PG cells and SA cells. Figure 1b illustrates the schematic structure of the artificial glomerulus. The axons of the ORNs expressing the same type of odorant in the olfactory epithelium make excitatory synapses with the MCs, the ET and the PG cells. The MC synaptically excites PG cell, which in turn inhibits the MC forming a negative feedback loop. The ET cells form excitatory connections with PG cells contributing indirectly to the inhibition of MCs. The connections across glomeruli are achieved through the SA cells, which receive inputs from ET cells and project their outputs into PG and ET cells. Note that ET cells along with SA cells form a sub-network that is isolated from MC and PG cells. So the activation of ET cells is determined only by the external input and the activity shared between them through SA cells.

Aungst et al. found that SAs in the OB play a critical role in the inter-glomerular connection, and, contrary to their name, they send interglomerular axons far away to form excitatory synapses with inhibitory PG neurons even up to 20–30 glomeruli away [16]. Considering these long-range connections, we provided our 16-glomeruli model with full connectivity between glomeruli. The network model is shown in Figure 1a where the full connectivity is represented by the thick red connection along the glomeruli. The model was implemented in MATLAB [27] using fourth-order Runge-Kutta ODE integration [28] with a time step of 0.1 ms. Initial conditions for all neurons were set to the resting potential.

### Objective Functions

Fisher's Discriminant Ratio [29], [30] and the Pearson's Correlation Coefficient [31] were used to provide a quantitative measure of the separability of odors and the correlation with odor concentration respectively. The Fisher's Discriminant Ratio *(FDR)* is defined as the ratio of the variance between classes and the variance within classes noted by
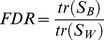
(4)


*S_B_* and *S_W_* being the between scatter and within scatter matrices, respectively, defined according to
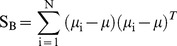
(5)

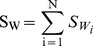
(6)

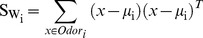
(7)where *x* are the experimental values or samples, *μ* is the mean value of all data, *μ_i_* is the mean of the samples of the *i^th^* odor, *N* is the total number of odor classes, and *Odor_i_* is the set of the samples of the *i^th^* odor. Following this definition, the *FDR* then increases proportionally to the separability of the classes, i.e. when the distance between the classes (*S_B_*) increases and, simultaneously, the dispersion of each class (S_W_) decreases. In our experiments, x is the mitral or tufted firing rate vector (one dimension per glomerulus) computed during the 0.5 second of odor presentation.

The Pearson's Correlation Coefficient *(PCC)* is defined as the level of correlation (or similarity) between two random variables calculated by
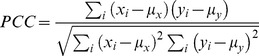
(8)where *μ_x_*, *μ_y_* are the mean values of all the samples of variables *x* and *y*, respectively, and *x_i_* and *y_i_* being the samples.

## Results

### Simulation Results

We have performed two experiments to determine how odorant information is segregated into identity and intensity in our computational model of the glomerular layer. In the first experiment, glomeruli were exposed to an odor stimulus dataset composed of six different odorants each one dosed at six different concentrations. In the second experiment, we tested the ability of the model to segregate odor information in the presence of an interfering odor. To do so, we reproduce the odor morphing experiments performed by [32] in rats where a sequence of binary mixtures going from one odor to another through intermediate mixtures was used (see Materials and Methods).

In both experiments we consider the mean activity of the principal neurons (MC, ET) during the experiment as the readout of the model. After an exposure of 0.5 seconds to each one of the 36 odors of the first experiment, we collect the output of MCs and ETs as a set of 36 16-dimensional vectors per each one of the output cells.

### Odors-concentrations experiment

**Figure 3:**
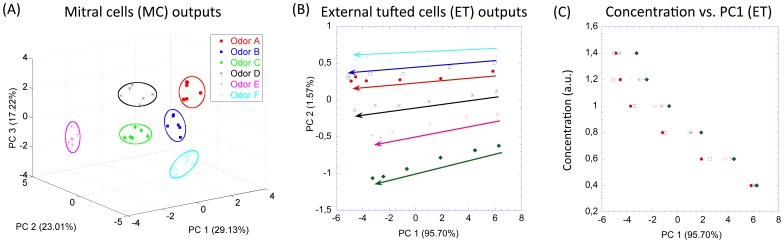
MCs and ET cells output in the 6 odors - 6 concentrations experiment. (A) Scores plot of the first three principal components of the MCs output. The output of MC is obtained as the mean firing rate during exposure of 0.5 s to the odors. (B) Scores plot of the first two principal components of the ET cells output. The output of ET cells is also obtained as the mean firing rate during the same experiment. Arrows indicate increasing concentration. (C) Scores of the first principal component of the ET output versus the stimulus concentration.

The exposure to six odors at six concentrations produced a data matrix of 36 (data) * 16 (glomeruli) for both the output of MCs and the ETs. We analyzed the results of this experiment using principal components analysis (PCA). Figure 3a shows the scores plot of the first three principal components of the MCs' output. This plot illustrates the two main processing outcomes of MCs: contrast enhancement and normalization of the input.

Specifically, the evident clustering of the odorants observed in Figure 3a disregarding odor concentration shows the normalization effect. On the other hand, the fact that odor clusters are better separated than the input data of Figure 2a shows the contrast enhancement effect of the MCs.

Specifically, the normalization effect is evidenced by the fact that odors are correctly clustered irrespective of their concentration. It is worth to compare figure 3a with figure 2a showing the PCA of the input stimuli to the model. It is also interesting to note the differences in the explained variance in the PCA plots of input and output data. The more sparse distribution of variance in the MCs output indicates the un-correlation of data in the output space. This supports the contrast enhancement function of MCs.

Figure 3b shows the scores plot of the first two principal components of the ET cells' outputs. This results show that ET have the opposite behavior than MC, in the sense that odorants in the ET output space are more correlated than in the input space. In this particular case, the first principal component captures more than the 95% of the variance, indicating that it is possible to estimate odor concentrations along the first principal component. This correlation is clearly shown in Figure 3c where the scores of the first principal component of the ET output are represented versus the stimulus concentration. At the same time, it is more difficult to identify the different odorants.

A comparison of MCs and ET cells output signals indicates that the information about odor identity and odor concentration that was encoded together in the input stimuli (Figure 2a) is decomposed in the glomeruli layer. The output of MCs represents odor identity while odor concentration is found in the tufted cells output. The separation of information can explain also the different actual dimension of the output spaces of the two cells. Identification requires a large space to accommodate the different odors, probably according to some chemical proximity, while the quantification only requires a single direction where concentrations can be ordered.

To confirm this qualitative results obtained with PCA, we have quantified the identity and concentration coding ability of the glomerular layer using two objective functions: Fisher's discriminant ratio (FDR) and Pearson's correlation coefficient (PCC). The FDR measures the degree of separation of the representations for different odors taking into consideration two elements: first, how close the representations belonging to the same odor are and; second, how far the representations of different odors are from each other. Thus, FDR allow us to measure the encoding of the identity of the odor. On the other hand, PCC give us the correlation between the representations for different concentrations of certain odor and the actual concentration value. Therefore, PCC quantifies the ability of encoding for odor concentration.

We computed the objective functions for the MC and ET outputs as a function of the synaptic efficiency of the SA. The synaptic connection of the SA is a key parameter that mediates the spread of activity across glomeruli. This parameter balances the contribution to each glomerulus of the external input and the lateral input from other glomeruli. For small values of the parameter, the external input dominates resulting in almost isolated glomerulus, whereas for large values of the parameter the lateral interaction dominates.

**Figure 4:**
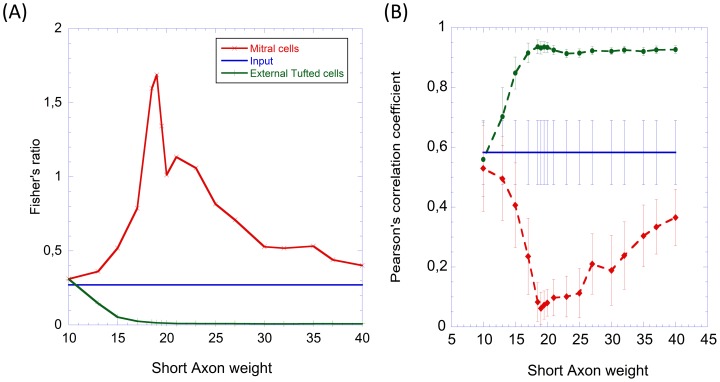
Quantitative measure of odor identity and concentration information in the 6 odors - 6 concentrations experiment. (A) Fisher's discriminant ratio of MC outputs, ETs cells outputs and input odor patterns in the 6 odors - 6 concentrations experiment. It is computed for different values of the short axon cell synaptic weights, which regulates the connection strength between glomeruli. (B) Pearson's correlation coefficient between MC, ET outputs, input odor patterns and the input odor concentrations for different short axon cell synaptic weights. The error bars show the standard deviation of the Pearson's correlation coefficient across different odors.

Figure 4a shows the FDR for MC and ET versus the SA synaptic weight. The FDR of the MC starts at a value close to that of the input stimuli for a synaptic connection weight of 10. It increases afterwards reaching a maximum in 19 and dropping subsequently until values close again to the input FDR. A small inhibition makes the glomerulus independent; therefore, the discrimination of the MC output is similar to that of the receptor layer. As the lateral interaction increases the inhibitory effect via PG cells modulates the output of the MC to cluster together odors at different concentrations. When the SA weight is greater than 19, the inhibitory effect becomes too large and a reduction of the activity of MC is observed. As a consequence the separation of odors decreases. The behavior of the FDR of the ET is simpler. Starting from a value similar to that of the input, the FDR degrades rapidly to reach a null value beyond 19. A strong lateral interaction enforces the contribution of the mean activity across glomeruli with respect to the specific activity received from the external stimuli by each glomerulus.

The correlation of MC and ET outputs with respect to the SA weight is shown in figure 4b. As in the FDR analysis, the results of the MC present an inflexion point for a SA weight value of 19. It reaches a minimum of correlation with the input concentration. Whereas the correlation of the ET cell responses with odor concentration sharply increases with the short axon weights up to an equilibrium stage. In particular, after a SA synaptic weight of 19 the PCC becomes almost completely independent from the SA weights.

These results clearly demonstrate that for SA weight of 19 [a.u.], the separability of the odor classes in the mitral cell output is maximized while simultaneously picking up the correlation between the ET cell's output and the odor concentrations. The FDR sharp maximum point shown in Figure 4a also explains the importance of the inhibition for the discrimination task.

### Morphing Experiment

In the morphing experiment, we expose the glomerular layer model with a series of 21 binary odor mixtures. This series of mixtures evolves from pure odor C (odor 1) to pure odor E (odor 21) going through 19 intermediate mixtures of both odors that slowly change from odor C to odor E (Figure 2b). The outputs obtained in the morphing experiment have been conveniently analyzed with hierarchical cluster analysis based on k-means [33]. This clustering method allows us to study the structure of the high-dimensional MCs and ETs output space in terms of the proximity of the different odors. Particularly, this algorithm performs an iterative clustering that provides a hierarchy of clusters. It starts clustering single odors and it ends up with a single cluster where all odors are merged. In this experiment we used the morphing odors along with the set of 36 combinations of odor-concentrations of the first experiment for comparative purposes.

**Figure 5:**
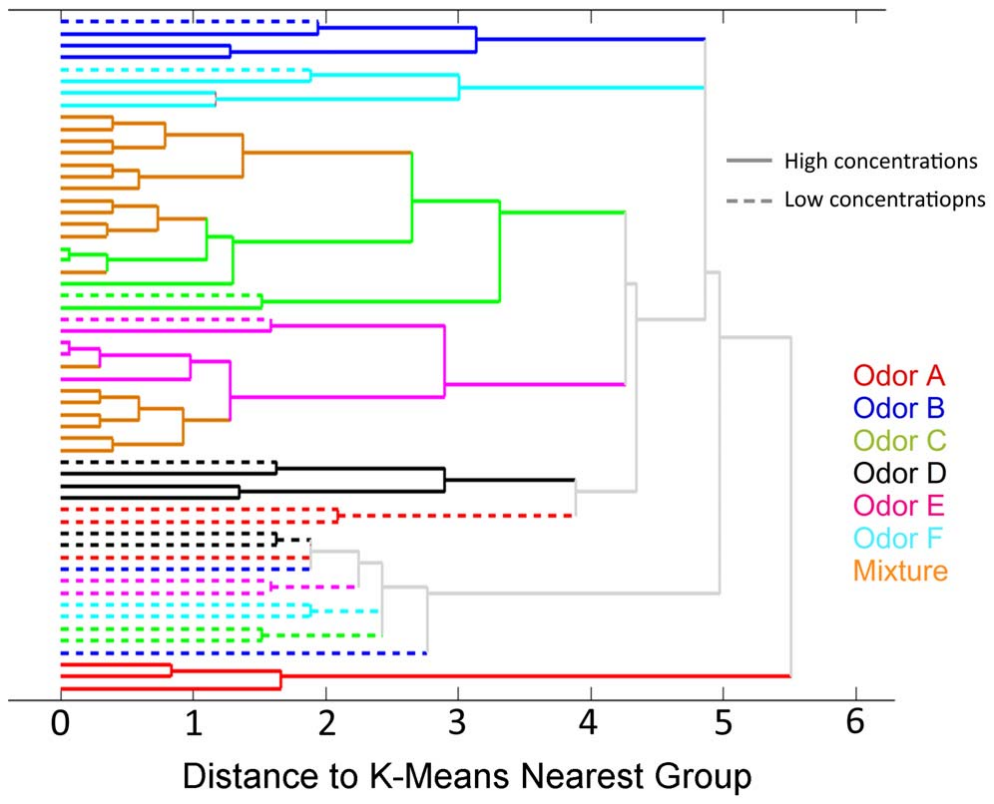
Hierarchical clustering of input patterns in the odor morphing experiment. We performed hierarchical clustering based on k-means on a sequence of binary mixtures going from odor C to odor E through intermediate mixtures (morphing) along with the odor patterns of the 6 odors - 6 concentrations experiment. Clustering results are presented as a dendogram in terms of the distance to the k-means nearest cluster. Odors are identified by color, where high concentrations are plotted in a solid line and low concentrations are plotted in a dashed line.

We applied first the hierarchical clustering to the input odors. The results are shown as a dendogram in figure 5. We can see that there is no evident grouping of the odors in the input space. Odors at different concentrations do not group all together. High (solid line) and low (dashed line) concentrations of odors do not seem to cluster together either. So clearly odor identity and odor concentration information are mixed up at the input space.

**Figure 6:**
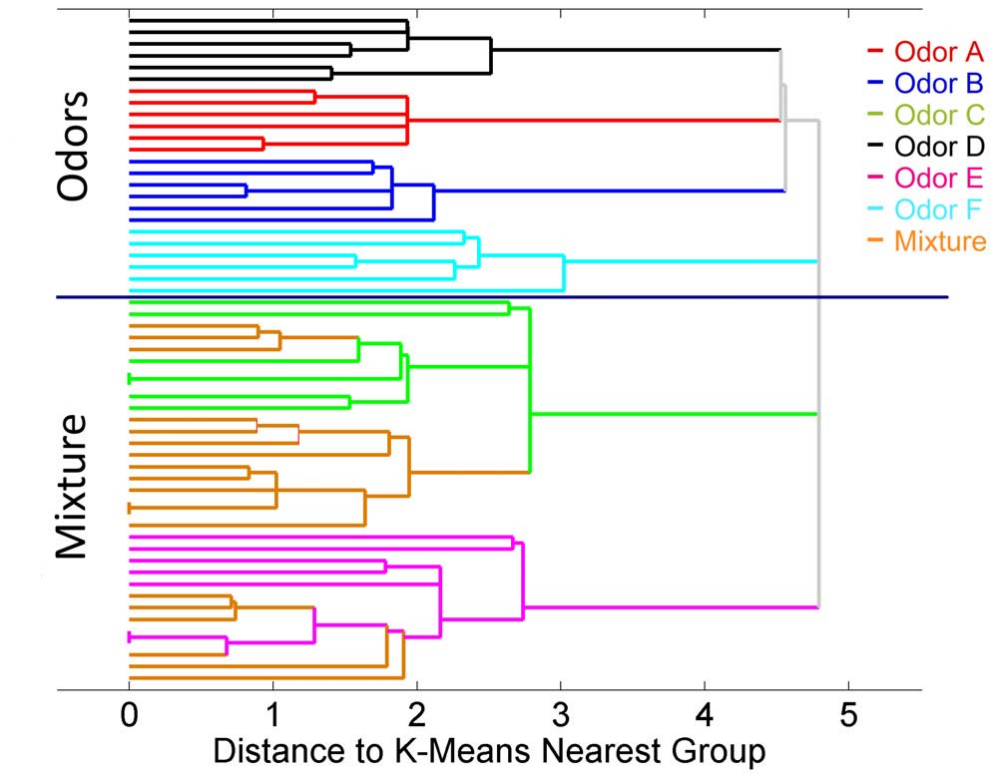
Hierarchical clustering of MC outputs in the odor morphing experiment. In this hierarchical clustering, MC outputs are grouped according to their identity in the case of pure odors. Mixtures cluster together and also with the different concentrations of pure odors C and E, which are the two components of the mixtures.

**Figure 7:**
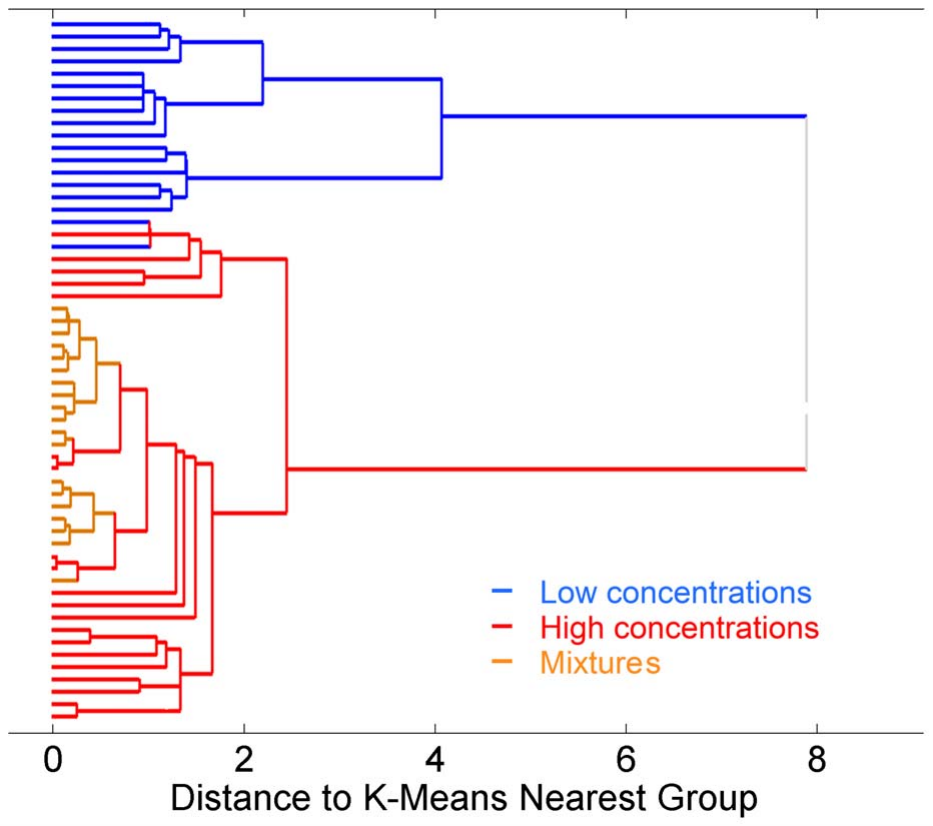
Hierarchical clustering of ET cells output in the odor morphing experiment. The hierarchical clustering of ET output patterns show a clear separation between high concentration odors (red lines) and low concentration odors (blue lines). Mixture odors lie within the high concentration cluster but not far from low concentrations. This is consistent with a proper disposition of concentrations since mixtures are formed by two components of concentration factors of 1 multiplied by mixing factor that sum up to 1 in all cases.

**Figure 8:**
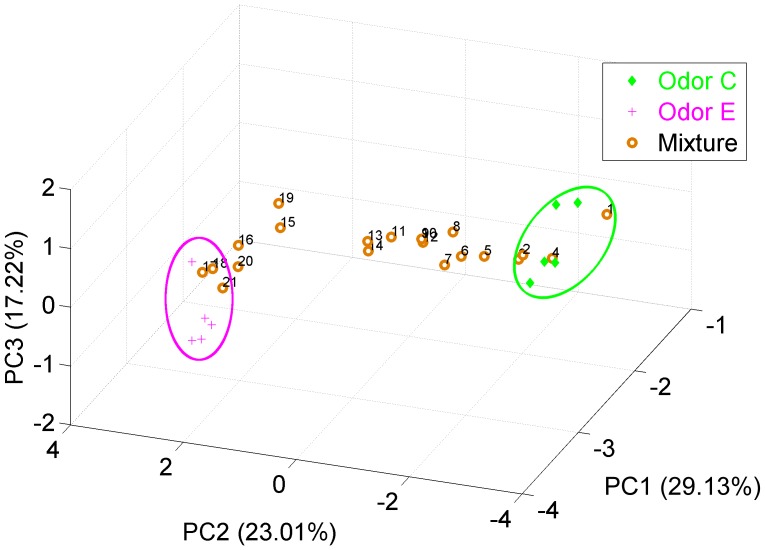
Smooth evolution of mixture odors in the morphing experiment. The figure represents the score plots of the 3 principal components of the MCs outputs in the morphing experiment. The 21 mixtures evolve smoothly from the initial odor C to the final odor E.

Figures 6 and 7 show the hierarchical clustering obtained for MC outputs and ET outputs respectively. The results show that MCs and ET cells naturally managed to group the data in clusters according to their odor identity and concentration. Specifically, the dendrogram of Figure 6 illustrates how MCs grouped the six pure odorants into six clearly identifiable clusters. Additionally, the binary mixtures are partitioned into the clusters corresponding to the two component odors, demonstrating once again that the MC outputs are highly correlated with the odor identity. Also note that mixtures are not grouped as new odors, but rather as the more abundant odor component in the mixture. This outcome of our study is consistent with the psychophysical experimental results obtained by Uchida et al. [3]. Finally, the morphing series allows us to determine that the MC output to odor mixtures evolve slowly from odor C to odor E. This is clearly observed in Figure 8 where the PCA scores of MC outputs show the transition of the mixtures from the cluster of one odor to the other cluster. This behavior reproduces the experimental results obtained by Khan et al. [32] in rats. Additionally, other groups have performed morphing experiments with either similar [34] or slightly different results [35]. Figure 7, on the other hand, shows that tufted cells separate data into groups according to the odor concentration independently of the odor classes. This behavior is more noticeable when considering the morphing data; in this case, the total concentration of the mixture is kept constant whereas the odor identity progressively changes from odor C to odor E, once again, the ET cells manage to classify the whole set of the morphing data into the same concentration cluster.

## Discussion

In this work, we have investigated the processing of information related to odor identity and odor concentration in the glomerular layer of the olfactory bulb. The recognition of odors irrespective of their concentration while preserving odor concentration information is a fundamental task developed by the olfactory system. We have built a computational model of the first stage of the olfactory bulb to investigate its ability to segregate odor identity and odor concentration information. Our findings illustrate that the processing of odor information at the glomerular layer can be the origin of the olfactory system ability to identify different odorants while still preserving information about their concentration. This is achieved by means of its two principal neurons the MCs and the ETs that encode odor identity and odor concentration information respectively, projecting this information afterwards to higher brain areas of the olfactory pathway.

Previous work by Cleland et al. [5], [6] have already shown the ability of the glomerular layer to perform several odor information processing tasks such as contrast enhancement, activity normalization, and extending dynamic range. They have also proposed that to cope with the high dimensionality of the olfactory input space, along with the lack of clear olfactory primitives (basis), the olfactory bulb relies on non-topographic strategies that makes this architecture different to other sensory systems. Their work, however, do not address the important issue of odor information segregation in the way it is done in the present work.

One of the interesting results obtained in this study is that there exists a minimum SA weight value for the glomerular layer to perform odor information segregation. As for odor intensity information, ET cells are able to maintain the performance even for values of the SA weight larger that this minimum. In the case of the odor identity information in the MCs, this value is a maximum beyond which the glomeruli loose their ability to capture odor identity. This behavior can be understood looking at the network architecture of the glomerular layer. The subnetwork of excitatory ET → SA → ET → SA is isolated from the rest of the glomerular network and receives inputs exclusively from ORNs. As a consequence, it shares the intensity level of all input ORNs among ET cells. So the stronger the link between ETs and SAs the better ETs capture the intensity information of the odor, which has contributions from all ORNs. The network that captures odor identity is more complex since we have the inhibitory effect of PG cells over the MCs. This inhibition is what mediates the normalization effect of the model by removing the concentration level from the MC response. The MC response is a balance between the excitatory effect of ORNs and the inhibitory effect of PG cells, which in turn are excited by ET and PG (odor intensity information). This is why the SA weight is critical and beyond certain value the inhibitory effect is too strong and the MCs responses do not capture odor identity so effectively. Another interesting result is that this minimum value of SA weight to obtain a maximum of performance is similar for odor intensity and odor identity. This could be an effect of using the same neuron model for MC and ET. However, this point has to be furthered studied.

Additionally, our results show that the glomerular layer is able to achieve odor segregation using the same neuron model (same parameters of Izhikevich's model) for MCs and ETs. These results demonstrate that the segregation of information in MCs and ET cells is due to network interactions and not to the different morphology of the projection neurons.

The information segregation capability of our model has been tested utilizing two different experiments; the first one involved a set of six odors at six concentrations, and the second involved a sequence of binary mixtures with composition slowly changing from one odor to the other. Our results in the first experiment conclusively demonstrated that the MCs portray information only about odor identity, whereas the ET cells' responses are much more correlated with odor concentration information (Figure 3a and Figure 3b). Notice that the scores of the tufted cells' output are stretched out along the first principal component capturing more than 95% of the total variance (Figure 3b). These different roles were confirmed in the presence of an interfering odor in the second experiment where a clustering method was used to study the structure of the MC and ET output space. Our results show that MC and ET can successfully segregate identity and concentration information with an odor mixture.

The morphing experiment allows us to validate our model with experimental results of neural activity in rats. Khan et al. [31] found that the response of rat MCs to a morphing sequence of binary odors is a neural representation that changes smoothly with the stimuli. Our results reproduce this behavior as shown in figure 8, where the MC output of the morphing series travels continuously from one of the components of the mixture to the other component. Uchida et al. [3] reported that rats are able to identify the more abundant component in a binary mixture. In this case two different classifications were obtained using the MC and ET cells' responses. The MCs' outputs allow classification of the input as a function of their identity; as a result, the data were naturally clustered as a function of the odor, and the mixtures were classified by taking into account the intensity of the dominant odorant in the mixture (Figure 6). Finally, the ETs' outputs separated the data as a function of their concentrations. Figure 7 clearly demonstrates that different odors at same concentration are classified in the same cluster.

Recent findings have unveiled new anatomical and physiological aspects of the glomerular layer. Liu et al. [36] found that the synaptic connection SA→ET is biphasic instead of been plain excitatory. The synapsis goes through a GABAergic inhibitory phase followed by a slower dopaminergic transmission. Gire et al. [37] have found that MC do not receive direct excitation from ORNs, instead they receive indirect external stimulus from ET cells. We have implemented these changes in our glomerular model to determine if the odor segregation ability is affected. Preliminary results show that the effect of the biphasic SA synapse is to synchronize the MC spikes whereas the global behavior is to degrade the odor segregation function. On the 6 odor 6 concentrations experiment, the FDR on Mitral cells goes from 0.50 in the unchanged model to 0.25 in the modified model (input odors 0.10). Furthermore, the PCC on ET drops from 0.82 in the unchanged model to 0.63 in the modified model (input odors 0.58). In any case, these are preliminary results and these modifications of the network need to be furthered studied.

In conclusion, the results obtained in this study are computational evidence that the architecture of the glomerular layer mediates the segregation of odor identity and odor concentration. This two pieces of odor information that are contained in the input stimuli are extracted and separated by the processing effect of the glomerular layer to provide odor identity information at the output of MCs and odor concentration information at the output of ETs. This neural mechanism may explain the ability of the olfactory system to recognize odors regardless of their concentration and at the same time identify their concentration level.
